# Localized aseptic neutrophilic dermatoses of the extremities triggered by stasis and tissue damage: Two case reports of an unrecognized condition misdiagnosed as bacterial cellulitis

**DOI:** 10.1016/j.jdcr.2022.07.036

**Published:** 2022-08-10

**Authors:** Valentina A. Imstepf, Christoph Schlapbach, Michael Benzaquen, Laurence Feldmeyer, Dan Lipsker, Luca Borradori

**Affiliations:** a Department of Dermatology, Inselspital, Bern University Hospital, University of Bern, Bern, Switzerland; b Dermatologic Department, University Hospitals of Strasbourg, University of Strasbourg, Strasbourg, France

**Keywords:** erysipelas-like rash, localized neutrophilic dermatoses, localized Sweet syndrome, lymphedema, ND, neutrophilic dermatosis

## Introduction

Neutrophilic dermatoses (NDs) are a group of autoinflammatory dermatoses characterized by an aberrant accumulation of neutrophil granulocytes in the skin with potential extracutaneous involvement and systemic inflammatory response.^1^^,^^2^ NDs often present with overlapping clinical and histopathological features, which led to the proposal of a simplified classification of NDs, encompassing superficial NDs, NDs en plaques, and deep NDs.^1^

NDs are typically associated with hematological disorders, chronic inflammatory, rheumatological, and gastrointestinal diseases. In addition to a variety of drugs, NDs may also be triggered or aggravated by traumas, including surgery.^1^

Here, we describe 2 unusual cases of localized NDs affecting the extremities, in which either acute or chronic peripheral edema resulted in a misleading inflammatory reaction that was invariably mistaken as cutaneous infection.

## Case reports

### Case 1

**Fig 1:**
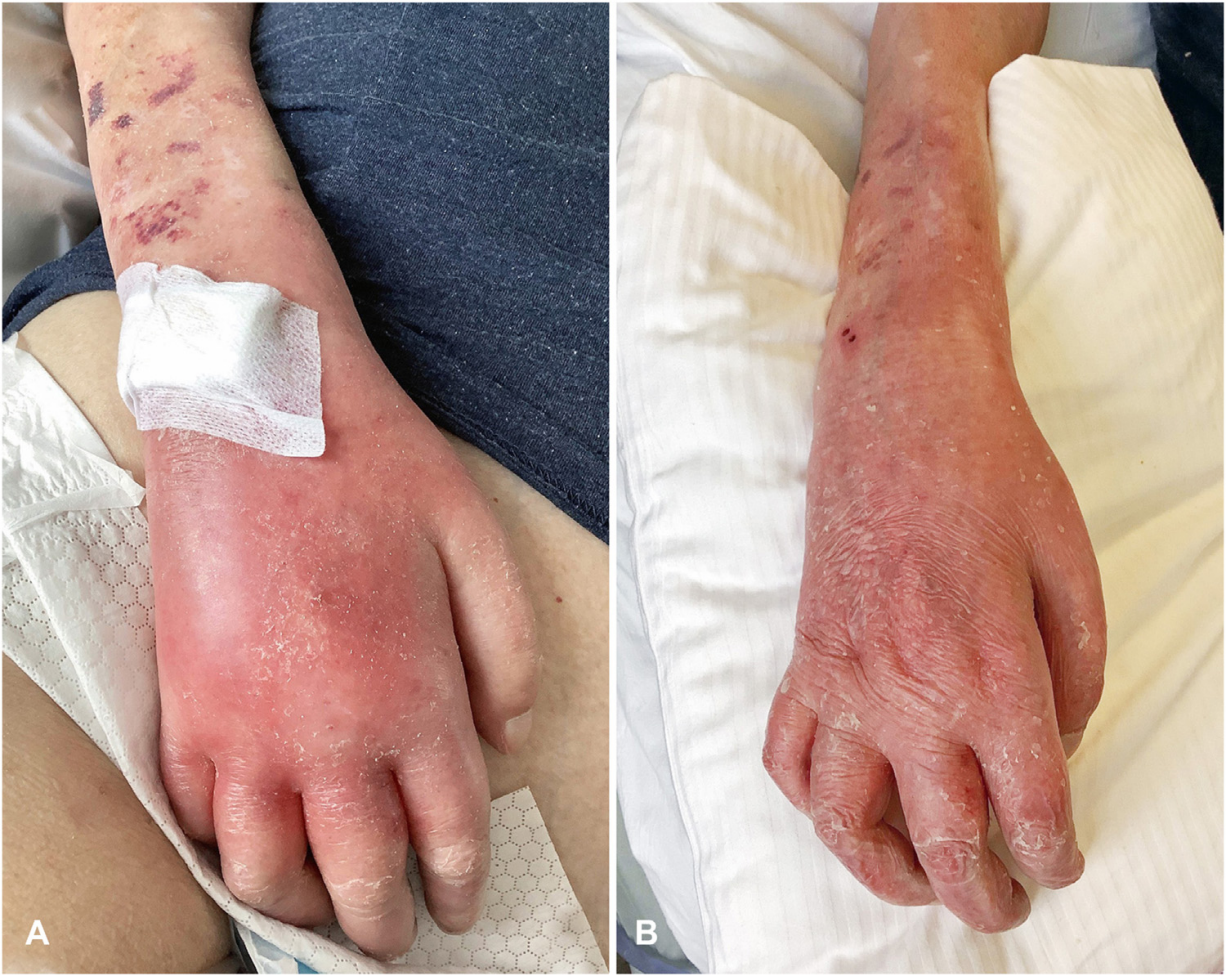
**A,** Symmetric acral involvement with an acute painful erythematous swelling of the dorsal surface of hands and fingers. **B,** Quick regression of the inflammatory syndrome and peripheral edema 5 days after treatment with topical clobetasol proprionate cream, empirical intravenous antibiotics, and protein-rich nutrition.

A 61-year-old man was referred to the emergency department for a systemic inflammatory syndrome. His past medical history was notable for untreated multiple sclerosis and chronic kidney insufficiency. Clinical examination revealed a pronounced edematous inflammation of the peripheral extremities (hands, fingers and feet) without any blisters or pustules (Fig 1, *A*).

**Fig 2:**
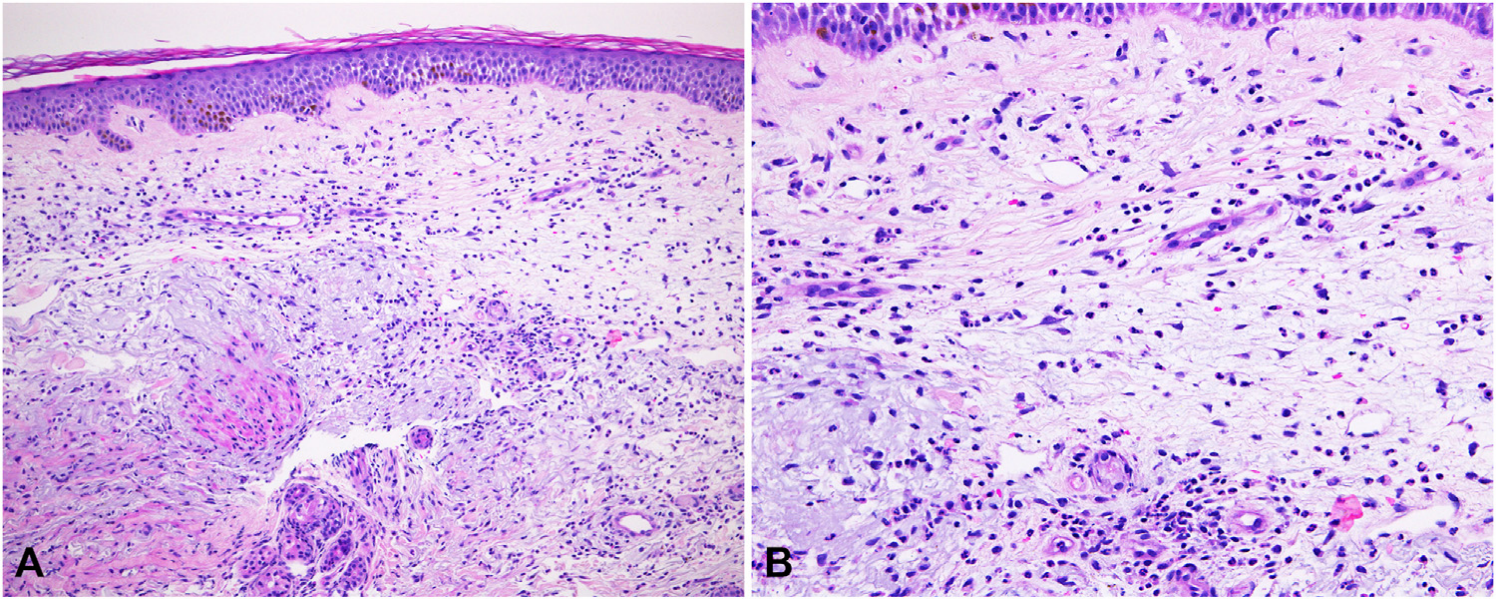
**A, B,** Light microscopy studies of a skin biopsy specimen obtained from the dorsal surface of the hand showing a diffuse and perivascular neutrophilic infiltrate, with leucocytoclasia and extravasation of erythrocytes in the entire edematous dermis with no evidence of vasculitis (**A** and **B**, Hematoxylin-eosin stain; original magnifications: **A**, ×100; **B**, ×200.)

Light microscopy studies of a skin biopsy specimen from the dorsal hand showed a diffuse and perivascular neutrophilic infiltrate, with leucocytoclasia and extravasation of erythrocytes in the entire edematous dermis with no evidence of vasculitis (Fig 2).

Laboratory tests showed an elevated C-reactive protein level up to 204 mg/L (normal, <5 mg/L) and an increased white blood cell count of 36 G/L (normal, 3-10.5 G/L) with 89% neutrophilic granulocytes.

Blood and urine cultures, polymerase chain reaction test for SARS-CoV-2, and microbiological culture of skin biopsy specimens remained negative. A severe hypoalbuminemia of 10 g/L (normal, 35-52 g/L) with microalbuminuria was explained by severe malnutrition and renal dysfunction.

The patient was treated with protein-rich nutrition, clobetasol proprionate cream and an empirically intravenous co-amoxicillin therapy for 5 days, allowing quick regression of the inflammatory syndrome and peripheral edema (Fig 1, *B*).

### Case 2

**Fig 3:**
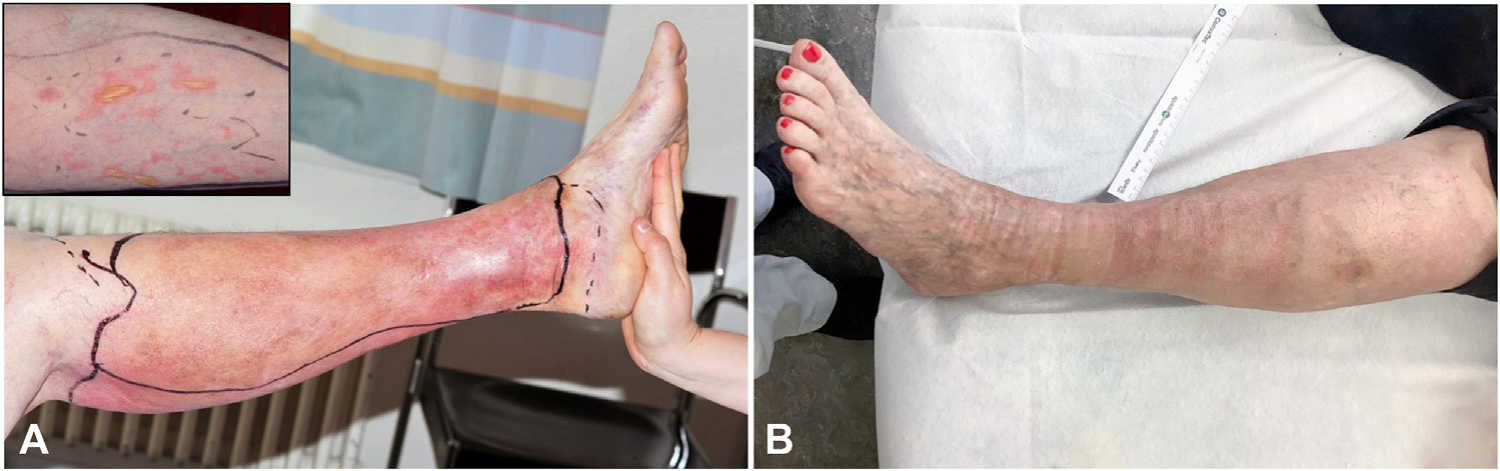
**A,** Painful swelling and redness in the left leg, warm and tender to touch, mimicking an erysipelas or a cellulitis associated with systemic symptoms and fever (temperature up to 40 °C). Erythematous papules and plaques with pseudobullous appearance on the left forearm (Inset). **B,** Complete regression of the clinical signs and the inflammatory syndrome after treatment with oral prednisolone. Signs of chronic venous stasis with varicose veins and pigmentation can be recognized.

A 47-year-old woman presented with an acute painful left red leg and fever (temperature up to 40 °C). She experienced at least 5 similar episodes during the previous year. The latter were unsuccessfully treated as bacterial cellulitis with different antibiotics, although pathogens could not be detected in the blood cultures. The patient suffered from chronic venous insufficiency because of multiple deep venous thromboses due to a factor V Leiden mutation, for which she was treated with acenocoumarol. On examination, she had an edematous, erythematous, warm, and painful left leg. She also showed erythematous papules and plaques with a pseudobullous appearance on the left forearm and abdomen (Fig 3).

**Fig 4:**
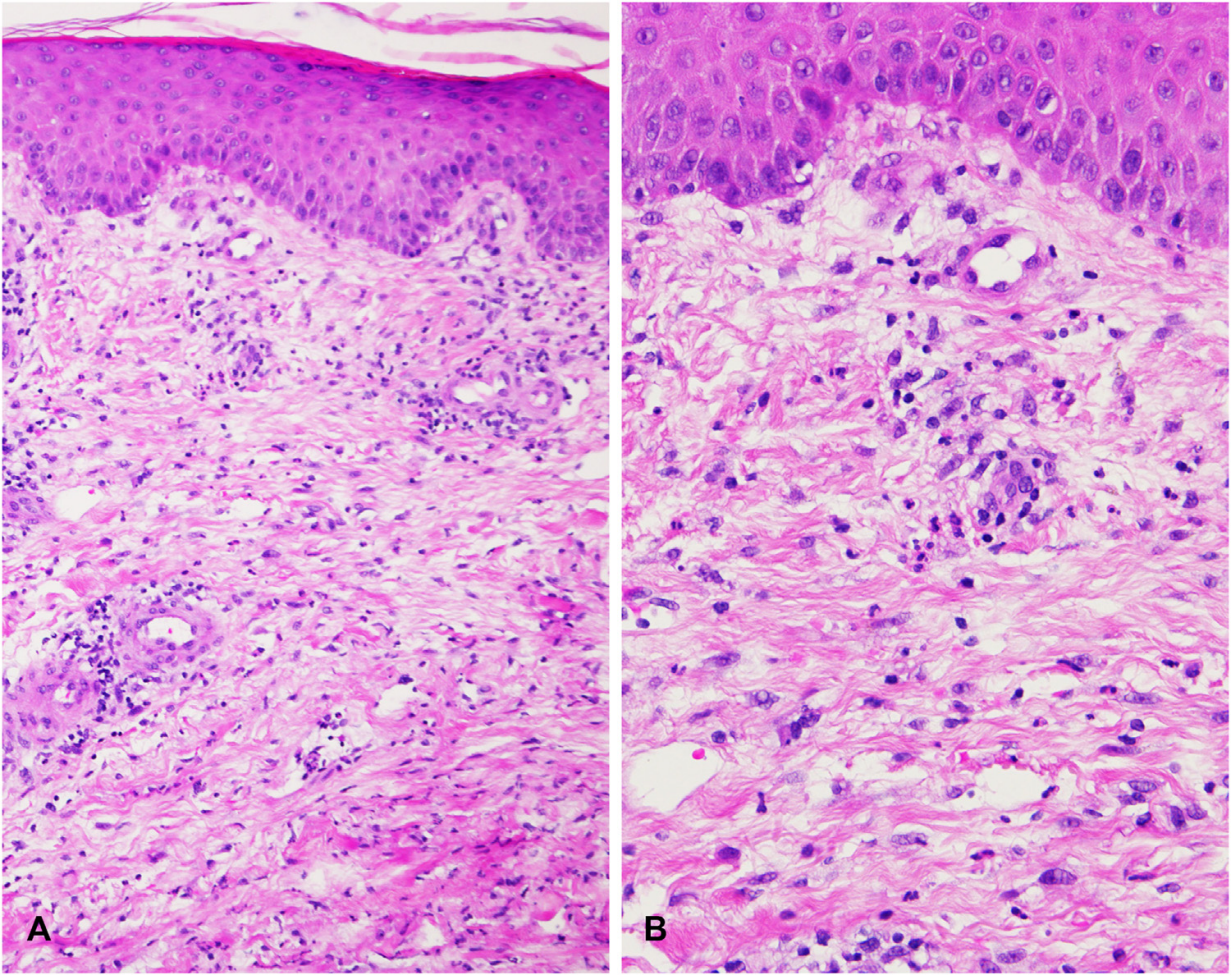
**A, B,** Light microscopy studies of a skin biopsy specimen obtained from the left leg demonstrating a strong dermal edema with superficial and deep neutrophil-rich infiltrate (**A** and **B**, Hematoxylin-eosin stain; original magnifications: **A**, ×100; **B**, ×200.)

Light microscopy studies of a skin biopsy specimen from the left leg showed strong dermal edema with a superficial and deep neutrophil-rich infiltrate (Fig 4).

Direct immunofluorescence microscopy studies remained negative. A computed tomography scan of the left leg showed no evidence of an underlying necrotizing fasciitis. A duplex sonography scan revealed no sign of deep venous thrombosis.

Laboratory tests found a C-reactive protein level up to 173 mg/L and neutrophilia of 20.5 G/L. Blood cultures remained negative. An extensive work-up did not find any evidence for either an extracutaneous organ involvement or an associated systemic disease, including a hematological malignancy.

The patient was treated with intravenous methylprednisolone 250 mg/d for 4 days, and then with oral prednisolone (0.75 mg/kg/d), with progressive tapering over 2 weeks. This regimen resulted in the complete regression of all cutaneous signs and the systemic inflammatory syndrome (Fig 3, *B*). The patient was then given colchicine 1.5 mg/d and, in the following year, she only had 2 mild episodes of red leg, which were controlled by a short course of oral prednisolone.

## Discussion

Our 2 observations represent striking inflammatory dermatosis associated with an inflammatory systemic response that closely mimicked acute bacterial infections. Although in 1 case, the acute eruption affected acral edematous areas, the second case was characterized by acute relapsing episodes involving an edematous leg related to chronic venous insufficiency. Although a search for underlying infectious pathogens remained negative in both cases and antibiotics showed little effect on the clinical course, both patients had an inflammatory dermatosis with histologically dermal edema with a neutrophil-rich infiltrate, which promptly responded to topical/systemic corticosteroids and anti-inflammatory drugs, such as colchicine.

Hence, these 2 cases should best be considered as autoinflammatory reactions triggered by a disturbance of the local homeostasis. We have recently described a series of patients with erythematous, inflammatory papules, plaques, and extensive infiltrated lesions, histologically characterized by a dermal neutrophil-rich inflammatory infiltrate that affected sites of surgery, chronic venous insufficiency, or lymphedema. Identical clinical phenotypes have previously been reported under a variety of confusing names.^3^, ^4^, ^5^, ^6^, ^7^

Therefore, we have proposed the unifying term of “localized NDs triggered by tissue injury” to better describe similar conditions that pose diagnostic and classification challenges.^5^ In our second case, the development of cutaneous lesions at distance from the primarily affected site suggests, that these localized NDs potentially spread under certain circumstances.

This question is clinically and economically relevant because of the significant incidence of erysipelas and bacterial cellulitis in the Western countries.^8^^,^^9^ The idea that some of these cases are incorrectly diagnosed as bacterial cellulitis and rather represent a localized ND is supported by 3 observations. First, some cases of these “infectious” dermatoses respond to simple anti-inflammatory regimens alone.^8^ Second, despite antibiotic prophylactic treatment, relapses surprisingly occur in more than 40% of patients. Finally, acute pseudocellulitis is a well-recognized condition that is frequently misdiagnosed and unnecessarily treated with antibiotics in the inpatient setting. Pseudocellulitis has been related in up to 30% of cases to venous stasis and is almost invariably mechanically explained by venous hypertension.^9^ Therefore, it is conceivable that both, rapid and chronic abnormal interstitial fluid and transcapillary fluid imbalance, result in tissue damage and local ischemia, leading to activation of damage-associated molecular pattern molecules with an inflammatory reaction,^5^ with cytokines activating neutrophilic leukocytes.^10^ In this setting, the presence of bacterial antigens, such as lipopolysaccharides, might also contribute to the inflammation.^2^

Better knowledge of these reactive, noninfectious disorders, which fulfill the criteria for an autoinflammatory disorder, is important to improve diagnostic accuracy and decrease hospital admissions and unnecessary antibiotic use.

## Conflicts of interest

None disclosed.
